# Functional Effects of Receptor-Binding Domain Mutations of SARS-CoV-2 B.1.351 and P.1 Variants

**DOI:** 10.3389/fimmu.2021.757197

**Published:** 2021-10-07

**Authors:** Rafael Bayarri-Olmos, Ida Jarlhelt, Laust Bruun Johnsen, Cecilie Bo Hansen, Charlotte Helgstrand, Jais Rose Bjelke, Finn Matthiesen, Susanne Dam Nielsen, Kasper Karmark Iversen, Sisse Rye Ostrowski, Henning Bundgaard, Ruth Frikke-Schmidt, Peter Garred, Mikkel-Ole Skjoedt

**Affiliations:** ^1^ Laboratory of Molecular Medicine, Department of Clinical Immunology, Section 7631, Rigshospitalet, Copenhagen University Hospital, Copenhagen, Denmark; ^2^ Recombinant Technologies, Novo Nordisk A/S, Måløv, Denmark; ^3^ Department of Infectious Diseases, Rigshospitalet, Copenhagen, Denmark; ^4^ Department of Clinical Medicine, Faculty of Health and Medical Sciences, University of Copenhagen, Copenhagen, Denmark; ^5^ Department of Emergency Medicine, Herlev and Gentofte Hospital, Copenhagen, Denmark; ^6^ The Blood Bank, Department of Clinical Immunology, Section 2034, Rigshospitalet, Copenhagen, Denmark; ^7^ Department of Cardiology, Rigshospitalet, Copenhagen, Denmark; ^8^ Department of Clinical Biochemistry, Rigshospitalet, Copenhagen, Denmark; ^9^ Institute of Immunology and Microbiology, University of Copenhagen, Copenhagen, Denmark

**Keywords:** SARS-CoV-2, RBD, ACE-2, B.1.351, P.1, variant of concern, E484K, N501Y

## Abstract

The recent identification and rise to dominance of the P.1 and B.1.351 SARS-CoV-2 variants have brought international concern because they may confer fitness advantages. The same three positions in the receptor-binding domain (RBD) are affected in both variants, but where the 417 substitution differs, the E484K/N501Y have co-evolved by convergent evolution. Here we characterize the functional and immune evasive consequences of the P.1 and B.1.351 RBD mutations. E484K and N501Y result in gain-of-function with two different outcomes: The N501Y confers a ten-fold affinity increase towards ACE-2, but a modest antibody evasion potential of plasma from convalescent or vaccinated individuals, whereas the E484K displays a significant antibody evasion capacity without a major impact on affinity. On the other hand, the two different 417 substitutions severely impair the RBD/ACE-2 affinity, but in the combined P.1 and B.1.351 RBD variants, this effect is partly counterbalanced by the effect of the E484K and N501Y. Our results suggest that the combination of these three mutations is a two-step forward and one step back in terms of viral fitness.

## Introduction

The continuous genetic drift resulting in immune adaptation of severe acute respiratory syndrome coronavirus 2 (SARS-CoV-2) has received international attention since the first identification of new emerging variants in the spring of 2020 (1). A specific focus has been on mutations in the spike gene, and in particular the residue changes that affect the receptor-binding domain (RBD, aa. 319–541) (2), responsible for the interaction with the human ACE-2 receptor (3–5). This ACE-2 interaction drives the transmission efficacy. Since most vaccine strategies are based on different spike-related immunogens (6), the RBD residue changes might pose major challenges in vaccine immune evasion capacity and receptor affinity adaptation (7–12).

Emerging new variants are being identified, monitored, and reported as a part of the international surveillance collaboration. Currently, there are 4 designated variants of concern (VOC) (Alpha/B.1.1.7, Beta/B.1.351, Gamma/P.1, and Delta/B.1.617.2) and 5 variants of interest (VOI) (Eta/B.1.525, Iota/B.1.526, Kappa/B.1.617.1, Lambda/C.37, Mu/B.1.621) (https://www.gisaid.org, https://www.who.int/en/activities/tracking-SARS-CoV-2-variants/, accessed 7^th^ September 2021). Interestingly, only relatively few positions in the RBD seem to be subjected to selective genetic drift. Some of the key residues involved directly in ACE-2 binding have evolved independently at different continents by convergent evolution. The consequences of these residue changes are being studied intensively and the growing VOC group, which includes to date the strains B.1.1.7 (Alpha), B.1.351 (Beta), P.1 (Gamma), and B.1.617.2 (Delta), has rapidly replaced the parent SARS-CoV-2 strain in the regions where they have been introduced. At the time of writing, new VOC/VOI are being reported almost every week (https://www.ecdc.europa.eu/en/covid-19/variants-concern, accessed 16^th^ June 2021). Nevertheless, a deeper molecular understanding of the interaction of the different strains with the host lags. All three VOC B.1.1.7, B.1.351, and P.1 contain the residue substitution N501Y due to an asparagine to tyrosine exchange in position 501 in the RBD domain. The N501Y substitution impacts transmissibility and disease severity (13–15). The VOC B.1.351, P.1, and B.1.617.2, and VOI B.1.525, B.1.617.1, B.1.620, and B.1.621 all have a glutamic acid residue at position 484 changed, which has been suggested to be a key residue for B-cell recognition and could thus affect the immunity level of vaccinated and convalescent individuals (16).

Here we present biophysical data of the impact of all the individual and combined residue changes in the RBD of P.1 and B.1.351 and show how these changes influence antibody neutralization of the ACE-2 interaction. We challenged the different constructs with sera from convalescent individuals (*n* = 150) previously infected with the parent Wuhan strain, sera from individuals after the 1^st^ and 2^nd^ dose with the BNT162b2 vaccine (*n* = 149), and a group of high-affinity monoclonal antibodies (*n* = 18) mapping to different epitopes on RBD.

The results show that the three residue substitutions in positions 417, 484, and 501 seem to have very different functional impacts on the RBD. The substitution of lysine^417^ to either an asparagine (K417N) or a threonine (K417T) results in a significant reduction in RBD/ACE-2 affinity, while the N501Y confers a ten-fold affinity increase towards ACE-2. Neither of these substitutions seems to have a major effect on the antibody neutralization from convalescent or vaccinated individuals. In contrast, the E484K displays a significant antibody evasion capacity without significantly impacting the affinity. Combined in the B.1.351 and P.1 RBD variants, the two different K417 substitutions also lower the overall affinity, but the effect is in part counterbalanced by the E484K and N501Y gain-of-function changes. Our results suggest that the combination of these three mutations is a two-step forward and one step back in terms of viral fitness related to ACE-2 affinity and antibody evasion capacity.

## Materials and Methods

### Production of Recombinant Proteins

The coding sequence of the SARS-CoV-2 RBD (QIC53204.1, aa **R319–S593**) was optimized regarding the codon adaptation index, 5’ mRNA folding energy, cryptic splice sites, polyadenylation signals, and tandem repeats as described elsewhere (17). Codon changes were introduced on the optimized sequence for the K417N, K417T, E484K, N501Y, and their combination K417N_E484K_N501Y (N_K_Y) or K417T_E484K_N501Y (T_K_Y). All sequences contained a C-terminal 10xHis-AviTag. The final sequences were synthesized and subcloned into pcDNA3.4-TOPO expression vectors by GeneArt (Thermo Fisher Scientific, Massachusetts, USA). A detailed description of the production and purification of all recombinant proteins, as well as the site-directed biotinylation of the RBD mutants, can be found elsewhere (12, 18).

### Determination of the Thermal Stability of the RBD Mutants

The thermal stability was analyzed on a Tycho NT.6 (NanoTemper Technologies GmbH, Munich, Germany) using the default 30°C/min thermal ramp. The RBD mutants were diluted to a final concentration of 0.5 mg/ml in PBS and analyzed in triplicates. Protein unfolding was monitored using the intrinsic fluorescence at 350 and 330 nm, and their ratio was used to determine the inflection temperatures (Ti).

### Determination of the RBD/ACE-2 Binding Kinetics by Biolayer Interferometry

Binding kinetics measurements were performed on an Octet RED383 system (ForteBio, California, USA) using the 16-channel mode with anti-human Fc capture (AHC) sensors (Pall Life Sciences, California, USA). A description of the experimental setup can be found elsewhere (12).

### ACE-2/RBD Antibody Inhibition Assay

The antibody-mediated inhibition potency of sera and mouse monoclonal antibodies (mAbs) was assessed using a previously reported ELISA-based ACE-2/RBD antibody inhibition test (a detailed protocol can be found elsewhere (18). Sera from COVID-19 convalescent individuals was analyzed at 10% serum dilution, while vaccine sera were analyzed at 10% (before and after the first dose) or 0.11% (after the second dose). The inhibition potency of sera (or neutralization index) was calculated as described elsewhere (18). The inhibition potency of mAbs was determined from 6-point 4-fold dilution series starting at 20 µg/ml and reported as logIC_50_.

### Blood Samples

The immune evasion potential of the RBD mutants was assessed in sera from 150 PCR-diagnosed COVID-19 recovered individuals [described elsewhere (19)]. The samples were randomly selected among those with a detectable antibody-mediated inhibitory response (estimated elsewhere (18)). The evasion potential was also evaluated in 149 randomly-selected serum samples from healthy individuals inoculated with the BNT162b2 vaccine that were collected before vaccination, approximately 2–5 weeks (range 13–33 days, median 23 days) after the first dose, and 2–8 weeks (range 11–53 days, median 34 days) after the second dose. The antibody-mediated inhibition potency was calculated using a serum pool from healthy individuals as the negative control. The Regional Ethical Committee of the Capital Region of Denmark approved the collection and use of blood samples (H-20028627 and H-20079890).

### Statistics

Statistical analyses were performed with GraphPad Prism 9 (GraphPad Software, California, USA, RRID : SCR_002798). Friedman test with Dunn’s multiple comparisons correction was used to analyze differences between the inhibition potency of convalescent and vaccinated sera by comparing the mean rank of each RBD mutant with the wt. Differences in the inhibition potency of convalescent sera and mAbs towards the RBD mutant were analyzed using two-tailed Spearman rank correlations (reported as correlation coefficient *ρ* and p-value *p*) and linear regressions (reported as goodness-of-fit *R^2^*). IC50 values for the panel of mAbs were interpolated from 6-point 4-fold serial dilutions using the equation [inhibitor] *vs* normalized response with variable slope. Non-inhibitory antibodies were normalized to 100.

## Results

### Individual B.1.351 and P.1 RBD-Defining Mutations Have Opposite Effects on Protein Stability and Binding Kinetics Towards ACE-2

**Figure 1:**
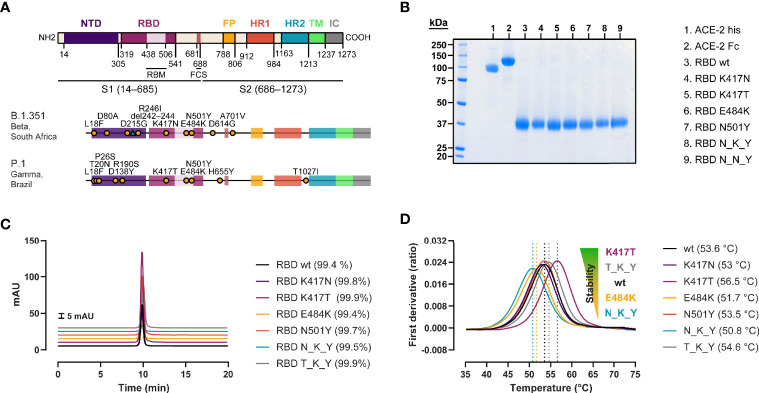
Generation of recombinant SARS-CoV-2 RBD variants with B.1.351 and P.1 RBD mutations. **(A)** Domain organization of the spike protein gene (22, 23), and location of the B.1.351 and P.1 strain-defining mutations (20, 21). NTD, N-terminal domain; RBM, receptor binding motif; FCS, furin cleavage site; FP, fusion peptide; HR1/2, heptad repeat 1/2; TM, transmembrane domain; IC, intracellular domain. **(B)** SDS-PAGE of the ACE-2 his-tagged and Fc-tagged used for the antibody-mediated neutralization assay and BLI measurements, respectively, and the biotinylated RBD variants under reducing conditions. **(C)** Purity determination by HPLC of the RBD variants. **(D)** Impact of the mutations on protein stability assessed by thermal denaturation experiments. Data are represented as the mean of the first derivative of the 350nm:330nm fluorescence ratio from 3 capillaries. Local maxima, signaled by vertical dashed lines, represent the inflection temperatures (Ti).

We produced recombinant RBD from SARS-CoV-2 wild type (wt, Wuhan strain), B.1.351 strain (originally identified in South Africa) (20), and P.1 strain (originally identified in Brazil/Japan) (21), as well as RBDs containing the individual mutations found in B.1.351 and P.1 strains (**Figure 1A**). The proteins were expressed in Expi293 cells and purified *via* their C-terminal His tag by IMAC coupled to SEC (**Figures 1B, C**). QC analyses revealed purities of > 99% as determined by HPLC-SEC for all the recombinant RBDs or ACE-2 proteins used in this study. The effect of the individual and combined mutations on the folding stability of the RBD was evaluated by thermal denaturation experiments (**Figure 1D**). When comparing the inflection temperatures (Ti) of the single RBD mutations with their wt counterpart, we observed a destabilizing effect for the E484K, and K417N mutations (−1.9 and −0.6°C respectively), and a stabilizing effect for the K417T (+2.9°C). This resulted in a net reduction of the thermal stability of the N_K_Y RBD (B.1.351 strain) (−2.8°C), and mild a net increase of T_K_Y RBD (P.1 strain) (+1°C). The N501Y had a very minor effect—if at all—on the Ti (−0.1°C).

**Figure 2:**
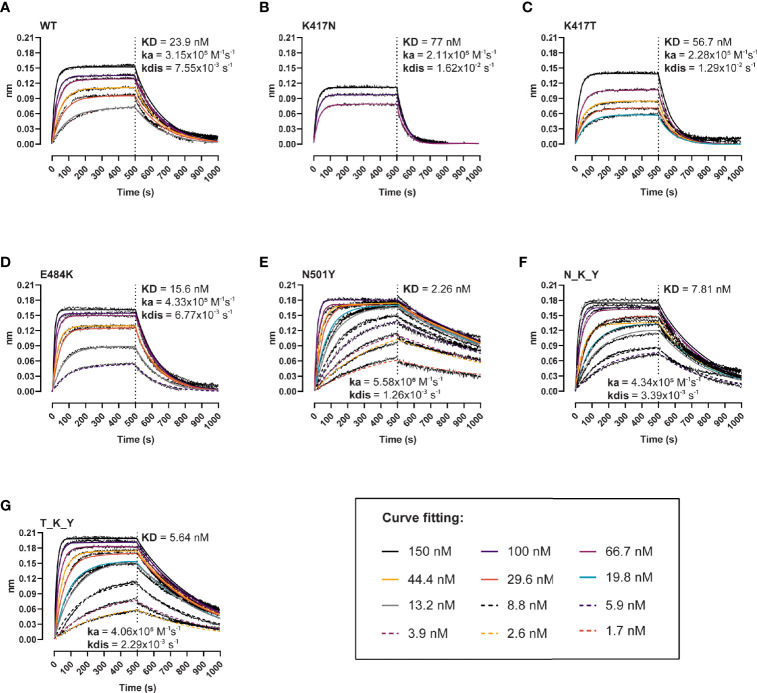
Binding kinetics of the interaction between human ACE-2 and the RBD variants. BLI binding response curves of RBD wt **(A)**, K417N **(B)**, K417T **(C)**, E484K **(D)**, N501Y **(E)**, B.1.351 (N_K_Y) **(F)**, and P.1 (T_K_Y) **(G)** to ACE-2-Fc immobilized unto AHC sensors. ACE-2-coated sensors were dipped into 12-point dilution series of RBD starting at 150 nM for 500 s, followed by a dissociation phase for 500 s. Colored lines represent global fits using a 1:1 binding model.

Next, we evaluated the effect of the individual and combined mutations on the binding kinetics of the interaction with the human ACE-2 receptor using BLI (**Figure 2**). The single residue changes in the 417 position resulted in two- to three-fold lower affinity (KD_K417N_ = 77 nM, KD_K417T_ = 56.7 nM, KD_WT_ = 23.9 nM) (**Figures 2A**–**C**), driven by faster dissociation rates (Kdis_K417N_ = 1.62x10^−2^ s^−1^, Kdis_K417T_ = 1.29x10^−2^ s^−1^, Kdis_WT_ = 7.55x10^−3^ s^−1^). The E484K variant provides a slight gain in affinity (KD = 15.6 *vs* 23.9 nM), association rates (4.33x10^5^ *vs* 3.15x10^5^ M^−1^s^−1^), and dissociation rates (6.77x10^−3^ *vs* 7.55x10^−3^ s^−1^) compared to the wt (**Figure 2D**), whereas the N501Y variant results in a ten-fold affinity increase (KD = 2.26 nM, ka = 5.58x10^5^ M^−1^s^−1^, kdis = 1.26x10^−3^ s^−1^). These combined and opposed effects are evidenced when analyzing the response curves of the B.1.351 RBD (N_K_Y) and P.1 RBD (T_K_Y) (**Figures 2F, G**), with binding parameters found between the ones of the RBD wt and N501Y: KD_N_K_Y_ = 7.81 nM, KD_T_K_Y_ = 5.64 nM, respectively.

### The E484K and N501Y Mutations Enhance the Evasion Capacity of B.1.351 and P.1 Against Natural-Induced Antibody-Mediated Immunity

**Figure 3:**
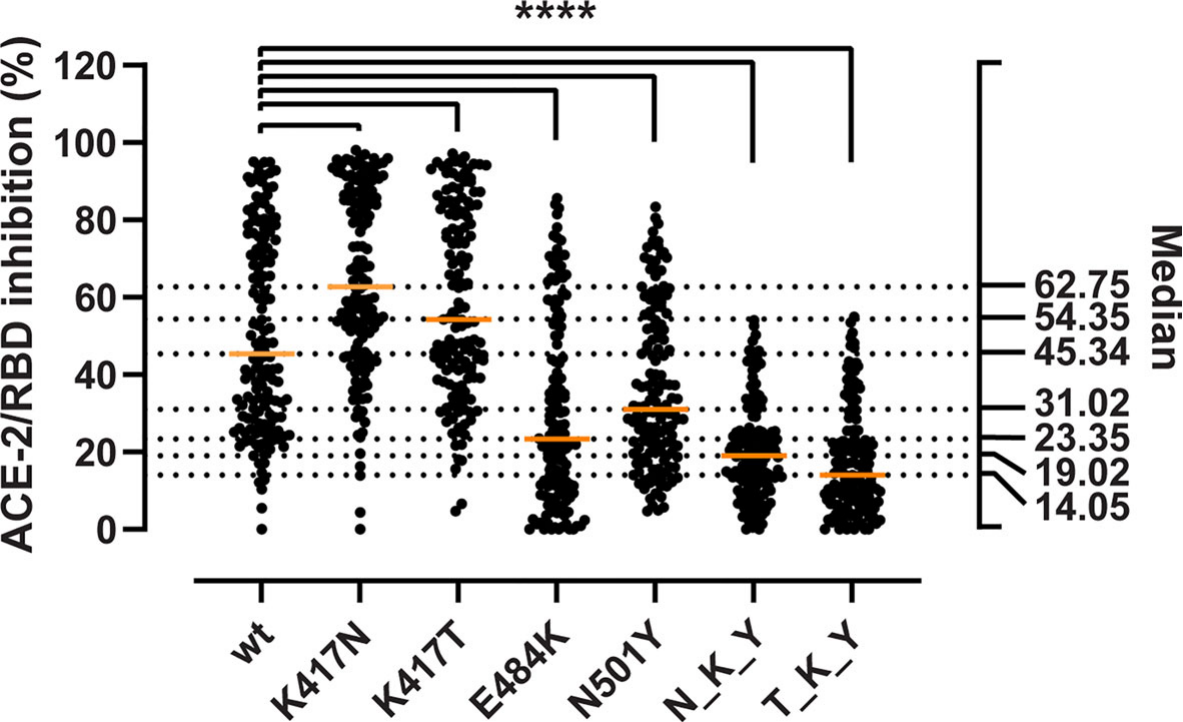
Inhibitory potency of COVID-19 convalescent patient sera against RBD variants. Antibody-mediated inhibition of serum from recovered COVID-19 patients (*n* = 150) against RBD wt, and the RBD-defining mutations from the B.1.351 and P.1 strains. Statistical comparisons between groups were performed using the Friedman test with Dunn’s multiple comparisons. Orange lines represent medians. The significance value applies to each of the pairwise comparisons between the wt and each of the single and combined mutations. ****, *p* < 0.0001.

We aimed at determining whether the mutations could enhance viral fitness beyond the considerable gains in the binding to the ACE-2 receptor. To do so, we evaluated their immune evasion potential with a validated antibody inhibition ELISA that measures the degree of inhibition (%) of sera or monoclonal antibodies of the ACE-2/RBD interaction (18). First, we determined the inhibition potency of sera from recovered COVID-19 patients (*n* = 150) (**Figure 3**). We observed a 1.9- and 1.5-fold reduction in the median inhibition of the E484K and N501Y RBD compared to the wt, while the substitutions in the 417 position had puzzlingly opposite effects with apparent 1.4- and 1.2-fold inhibition gain for K417N and K417T, respectively (Friedman test *p* < 0.0001 for all). When considered in combination, the B.1.351 RBD (N_K_T) resulted in a 2.4-fold reduction in the median inhibition potency, and the P.1 (T_K_Y) in an even more dramatic 3.2-fold reduction (*p* < 0.0001 for both). The inhibition potencies towards the wt RBD and the mutations were significantly correlated (*ρ* values ranging from 0.763 to 0.945, Spearman rank *p* < 0.0001) (**Supplementary Figure 1**).

### The RBD Mutations Have a Minor Effect on a Panel of Neutralizing mAbs

**Figure 4:**
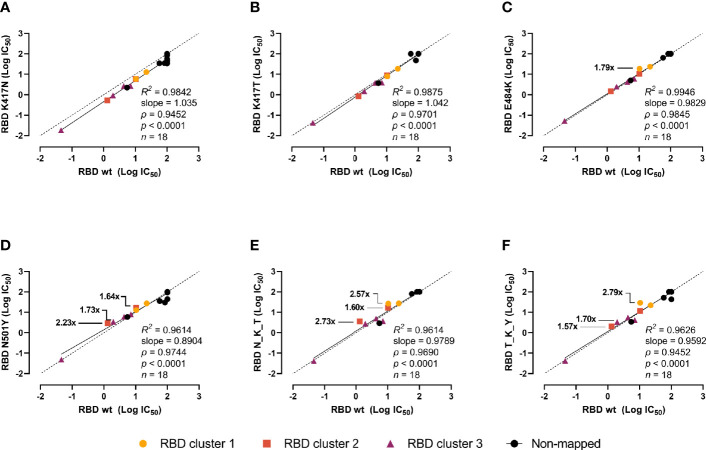
Impact of the RBD mutations in mouse mAbs-mediated neutralization. The inhibition potency of mouse mAbs (reported as logIC_50_) was determined by incubating the RBD variants with a 6-point 4-fold serial dilution of mAbs starting at 20 µg/ml. IC_50_ were calculated with the equation [inhibitor] *vs* normalized response with variable slope after normalizing to non-specific mAbs. Linear regression and Spearman correlation analyses of the logIC_50_ (*n* = 18) for RBD wt *vs* K417N **(A)**, K417T **(B)**, E484K **(C)**, N501Y **(D)**, B.1.351 (N_K_Y) **(E)**, and P.1 (T_K_Y) **(F)**. Solid line represents linear regression (goodness of fit reported as R^*2*^), dashed line represents equidistance between axes. Fold changes in the inhibition potency is shown only for reductions above 1.5-fold.

Next, we investigated whether the mutations could escape recognition by a panel of 18 high-affinity murine mAbs raised against wt RBD or the prefusion-stabilized spike ectodomain (18). The mAbs were divided into 4 groups (or clusters) based on previous epitope mapping analyses: 3 with epitopes in the RBD and varying inhibition potencies (clusters 1 to 3), and 1 with non-competing epitopes with clusters 1–3 and non-neutralizing (non-mapped). The RBDs were incubated with serial dilutions of the mAbs, and the interpolated IC_50_ values of the mutant RBDs were pairwise compared with the wt using linear regression and Spearman correlation analyses (**Figure 4**). The inhibition potencies of the individual mAbs correlated strongly (*ρ* > 0.96, *p* < 0.0001 for all). The K417N (**Figure 4A**) and K417T mutants (**Figure 4B**) had no noticeable effect. The E484K mutant had moderate effects on a mAb from cluster 1 (1.79-fold inhibition reduction) and N501Y on mAbs from clusters 3 and 3 (reductions ranging from 1.64- to 2.23-fold) (**Figures 4C, D**). This moderately impaired inhibition was also present in the N_K_Y and T_K_Y combined mutants (**Figures 4E, F**). Notwithstanding, none of the single mutation or combination of them escaped recognition by mAbs.

### The E484K Is the Major Determinant of the Evasion Capacity of the RBD Variants Against Vaccine-Induced Antibodies

**Figure 5:**
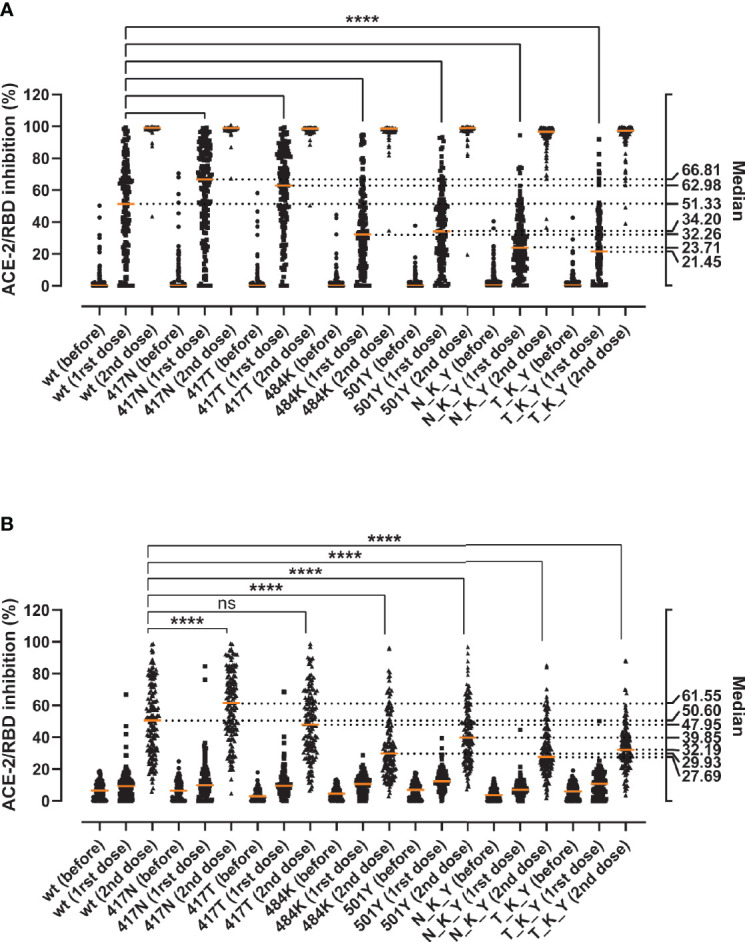
Inhibition capacity of sera from healthy individuals before and after the BNT162b2 vaccine against the RBD variants. Samples were collected immediately before (before), around 3 weeks after the first (1rst dose), and 5 weeks after the second vaccine dose (2nd dose) (*n* = 149). Statistical comparisons of the neutralization indexes for the different variants were performed at 10% serum after the first dose **(A)** or at 0.11% serum after the second dose **(B)** using the Friedman test with Dunn’s multiple comparisons. Orange lines represent medians. The significance value (**** or ns) reflects the pairwise comparisons between the wt and each of the single and combined mutations. Based on the visual and numeral evaluation of the spread of the data, pairwise statistical comparisons after the first dose were performed at 10% serum **(A)**, while differences after the second dose were evaluated at 0.11% serum **(B)**. ns, non-significant; ****, *p* < 0.0001.

Finally, we assessed the immune evasion potential of the variants in sera from individuals immunized with the BNT162b2 vaccine. We collected blood samples from 149 healthy individuals before vaccination, around 3 weeks after the first dose, and 5 weeks after the second dose. The antibody-mediated RBD/ACE-2 inhibition was determined in serial dilutions of serum to account for the marked inhibition differences of naïve and vaccinated sera (i.e. 10% for naïve sera and sera after the first dose, and 0.11% for fully vaccinated sera) (**Figure 5**). After the first dose, and as observed previously, the substitutions in the 417 position (417N and 417T) appear to be more effectively inhibited than the wt (median_K417N_ = 66.81, median_K417T_ = 62.98, median_wt_ = 51.33, Friedman test *p* < 0.0001) (**Figure 5A**). Both the E484K and N501Y alone and as part of the N_K_Y and T_K_Y significantly impaired the antibody-mediated inhibition (*p* < 0.0001 for all), ranging from a 1.5-fold decrease for the N501Y alone, to a 2.39-fold for the combined T_K_Y (medians 51.33 wt, 32.26 E484K, 34.2 N501Y, 23.71 N_K_Y, 21.45 T_K_Y). The inhibition capacity increased dramatically after the second dose (**Figure 5B**), albeit the relative differences in the inhibition capacity against the variants remained for the most part unchanged. The K417N—but not the K417T—appeared to be inhibited better than the wt. At the same time, the E484K and N501Y RBD still caused a significant decrease in the median inhibitory potency of fully vaccinated sera (1.7-fold and 1.3-fold decrease respectively) (*p* < 0.0001 for both). The effect of the N_K_Y and T_K_Y ranged from 1.6- to 1.8-fold inhibition potency decrease (*p* < 0.0001 for both), comparable to that of E484K alone.

## Discussion

The relationship between established immunity from either recovered COVID-19 disease or vaccination and new emerging genetic SARS-CoV-2 strains is being heavily studied and debated.

**Figure 6:**
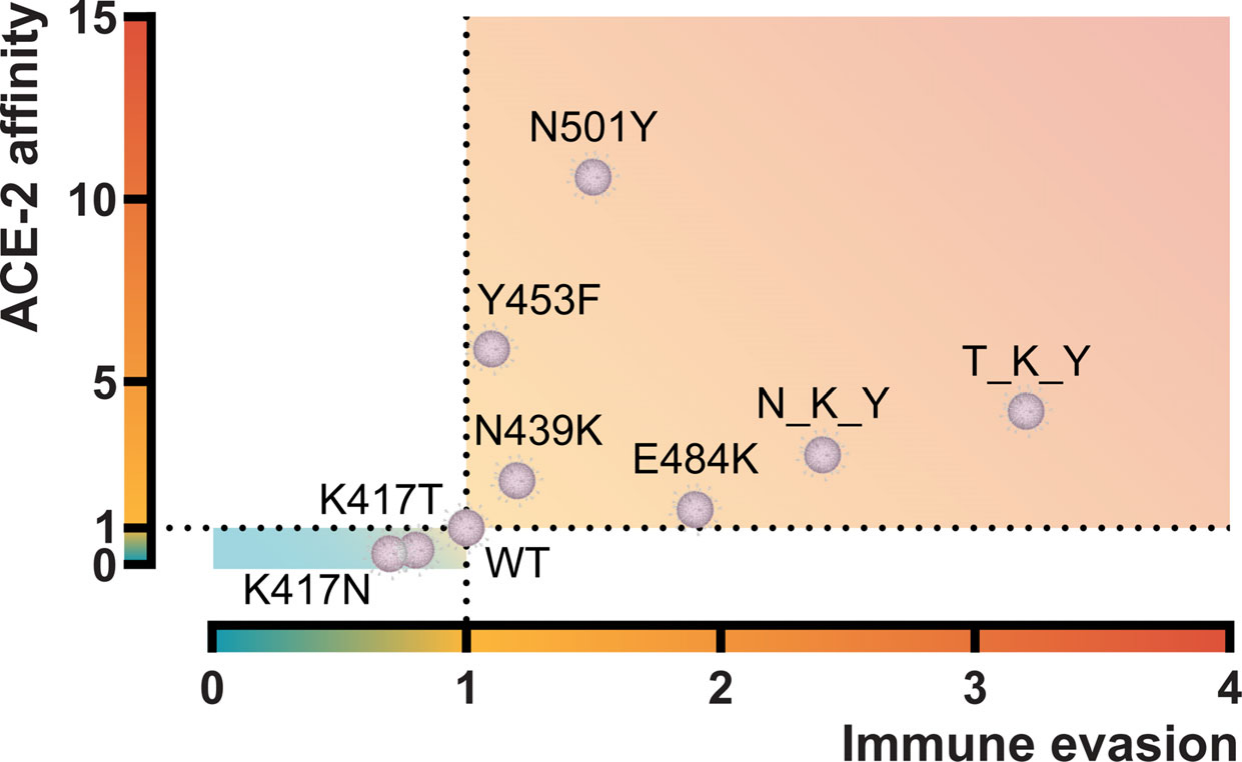
Graphical representation of the ACE-2 affinity and immune evasive capacity of RBD variants. ACE-2 affinity was calculated by BLI and represented as fold difference compared to the RBD wt. Immune evasion was calculated using an ELISA-based ACE-2/RBD antibody inhibition assay (18) and represented as the fold-difference of the median antibody-mediated inhibition of convalescent sera. N_K_Y, B.1.351 RBD (K417N + E484K + N501Y); T_K_Y, P.1 RBD (K417T + E484K + N501Y). Sources (12, 18) and unpublished data.

The confirmed worldwide COVID-19 cases are relentlessly reaching more than 220 million cases at the time of writing (https://www.worldometers.info/coronavirus) (24). Since the beginning of the pandemic, many single residue substitutions and deletions have been reported in the SARS-CoV-2 spike molecule. However, the changes in the receptor-binding domain remain are relatively limited. Indeed, comparative genomics of 44 Sarbecovirus strains revealed fewer-than-expected mutations in the S1 (which harbors the RBD), suggesting a recent adaptive deceleration (25). This might suggest that SARS-CoV-2 has a restriction in the “mutational degree of freedom” for the domains and sites involving interaction with human ACE-2. The new dominating variants that seem to take over in different regions of the world appear to drift in many of the same RBD positions. These residue substitutions have been shown to improve the overall viral fitness. In this study, we focused on characterizing the two variants first described in South Africa (B.1.351, Beta) and Brazil (P.1, Gamma) and the impact of both the single and combined residue changes in the RBDs on ACE-2 affinity and antibody derived immune evasion from convalescent or vaccinated individuals and monoclonal antibodies. These results and those from other RBD variants characterized by our group have been summarized in **Figure 6**.

When we assessed the impact of the individual residue substitutions, we found that the N501Y increased the affinity towards ACE-2 ten-fold, whereas the opposite was the case for the lysine substitution at position 417 to either a threonine or an asparagine residue that resulted in a reduced affinity of around three-fold compared to the original wt. The E484K did not influence the affinity significantly but had a pronounced increased antibody evasive capacity. Our findings are in agreement with other recent reports (8, 26–30). Surprisingly, both convalescent and vaccinated individuals (originally exposed to either the “wild-type” Wuhan strain or the mRNA sequence that translates into wt spike) mediated a better neutralization of the K417T or K417N RBD/ACE-2 compared to wt. We are not aware of reports showing that 417 variations exist independently. However, because the amino acid exchanges in position 417 decrease viral fitness by impairing the interaction with ACE-2—based on our experiments, it is pertinent to speculate that mutations in positions 484 and 501 may have been driven by positive selection to counterbalance the effect of the 417 substitutions. The N501Y mutation has been reported in mice to emerge as adaptation after serial passaging of a SARS-CoV-2 clinical isolate and increase its virulence (31). Yeast surface *in vitro* evolution experiments aimed at increasing ACE-2 affinity have shown that the E484K and N501Y were among the first mutations to be selected and fixed (32). Moreover, the authors showed that adding the K417T/N mutation into an E484K/N501Y background increased the surface expression of the triple mutant, suggesting positive cooperativity between these three mutations. It is also possible that the K417T/N substitution arose by genetic drift after the fixation of the E484K and N501Y mutations and may thus be regarded as a “neutral passenger” during the development of viral diversity. It has been proposed that the unusual accumulation of mutations in the spike protein in the B.1.351 and P.1 variants may have been the result of within-host evolution in an immunocompromised individual (33–35). It has also been noted that the 417 position is part of a perfectly conserved region among sarbecoviruses, with the notable exception of SARS-CoV-2 and its close bat homolog RaTG13 (25). The authors suggested that this position might have changed to a non-optimal lysine in the SARS-CoV-2/RaTG13 common ancestor by genetic drift, and as such, it is less constrained and more likely to mutate. This is supported by experimental data from deep mutational scanning analyses of the SARS-CoV-2 RBD that revealed that the 417 position has high entropy, i.e. lower mutational constraints (8). The Indian government has recently reported the new variant form of the B.1.617.2 (Delta) VOC to have stronger binding to receptors of lung cells and a reduction in the monoclonal antibody response (36). In light of our results, we would not expect the Delta plus to provide any advantage compared to the Delta in terms of binding affinity or immune evasion capacity. However, we should exercise caution when directly translating *in vitro* findings derived from purified proteins and protein domains into an *in vivo* host/viral interaction setting.

Antibody neutralization of the RBD/ACE-2 interaction will constitute a diverse polyclonal pool of individual antibodies with different epitopes and varying affinity. Thus, the inhibition level will be influenced both by the number and quality of the antibodies, but also by the actual RBD/ACE-2 affinity. Therefore, the enhanced neutralization of the single 417 RBD constructs could result from the lower K417N/T-RBD/ACE-2 affinity that might allow for a larger pool of RBD antibodies to bind with lower affinity in the 50–70 nM range. Conversely, the decreased neutralization of the N501Y mutation observed in our RBD/ACE-2 inhibition assay may stem from a combination of compromised recognition by neutralizing antibodies and the higher affinity RBD/ACE-2 interaction outcompeting antibodies with affinities around the RBDwt/ACE-2 Kd, and thus displacing the fluid-phase equilibrium towards ACE-2-bound RBD and away from antibody-bound RBD. To date, the extent to which the N501Y mutation challenges established immunity remains under debate. Several studies have shown that the N501Y compromises neutralization by many mAbs (37, 38), but polyclonal convalescent and vaccine sera remain, for the most part, effective at neutralizing the N501Y-containing B.1.1.7 (alpha) variant (39). It also needs to be taken into account that while the N501Y is the only RBD mutation in the B.1.1.7 variant, the latter carries other changes in the spike protein—such as deletions in the NTD—that may contribute to its immune evasion properties (40, 41).

Single residue substitutions are normally unlikely to challenge a distributed polyclonal B-cell response, but we observe a significant evasive capacity of the E484K and combined with the N501Y driving a higher RBD/ACE affinity in the low nM range it might challenge established immunity at a low maturation state. On the other hand, when we challenge the different variants with a variety of different matured high-affinity monoclonal antibodies (*n* = 18) belonging to different epitope clusters, we did not observe a dramatic difference in the neutralization capacity between the variants tested here. Combined, the results thus suggest that the virus neutralization is not just a matter of changes of key residues in immunodominant B-cell epitopes but could also be a balance between the B-cell affinity maturation state and the biophysical affinity of the receptor affinity adapted strains. Interestingly, the same combined E484K/N501Y is found in the VOI B.1.621, originally identified in Colombia (42), and the P.3 (Theta), identified in the Philippines (43), but without the 417T or N substitution that likely reduces the overall virus fitness. Functional data is not present now, but time and further studies will tell if these variants might be of even greater concern than the B.1.351 and P.1 strains examined in this study. Two other interesting substitutions are found in the RBD of two distinct lineages within the B.1.617 variant (B.1.617.1/B.1.617.3) originated in India (44), where the glutamic acid in position 484 is changed to glutamine (E484Q) that might drive an evasive potential. Instead of carrying the N501Y, all lineages within the B.1.617 variant include a leucine to arginine substitution at position 452 (L452R), that have been shown to increase affinity towards ACE-2, as well as infectivity and resistance to antibody-mediated neutralization *in vitro* (45, 46). However, the situation might be much more complicated in a physiological setting due to the interaction valency between the virus and the target cell and the clonality, distribution, and accessibility of antibodies present in the alveolar lumen space.

Taken together, we have characterized the individual and combined residue substitutions in the RBD of the P.1 and B.1.351 variants. We can show that they are a combination of gain- and loss-of-function in terms of affinity and antibody-mediated evasion. A particular focus should be on the 417 position, which seems to be a mutational hot spot, where a gain-of-function residue change might result in even greater virulence.

## Data Availability Statement

The original contributions presented in the study are included in the article/**Supplementary Material**. Further inquiries can be directed to the corresponding author.

## Ethics Statement

The studies involving human participants were reviewed and approved by The Regional Ethical Committee of the Capital Region of Denmark approved the collection and use of blood samples (H-20028627 and H-20079890). The patients/participants provided their written informed consent to participate in this study.

## Author Contributions

RB-O, PG, and MOS performed conceptualization (idea conception and study design); RB-O, CH, FM and JRB performed investigation (recombinant protein design, production, and purification); LBJ performed investigation (RBD/ACE-2 binding kinetics characterization by BLI); RB-O and IJ performed investigation (ELISA-based experiments); CBH, SDN, KKI, SRO, HB, and RF-S performed resources (provision and registration of blood samples); RB-O performed formal analysis; RB-O, MOS, and PG performed writing – original draft. All authors contributed to the article and approved the submitted version.

## Funding

This work was financially supported by grants from the Carlsberg Foundation (CF20-0045) and the Novo Nordisk Foundation (NFF205A0063505 and NNF20SA0064201).

## Conflict of Interest

LBJ, CH, JRB and FM were employed by Novo Nordisk A/S.

The remaining authors declare that the research was conducted in the absence of any commercial or financial relationships that could be construed as a potential conflict of interest.

## Publisher’s Note

All claims expressed in this article are solely those of the authors and do not necessarily represent those of their affiliated organizations, or those of the publisher, the editors and the reviewers. Any product that may be evaluated in this article, or claim that may be made by its manufacturer, is not guaranteed or endorsed by the publisher.
