# Supplementary material for: Functional Effects of Receptor-Binding Domain Mutations of SARS-CoV-2 B.1.351 and P.1 Variants
Source: Front Immunol. 2021 Oct 7;12:757197. doi: 10.3389/fimmu.2021.757197 (PMC8529273; doi:10.3389/fimmu.2021.757197)
Supplement: Supplementary file 1 [file DataSheet_1.docx]

Supplementary Material

**
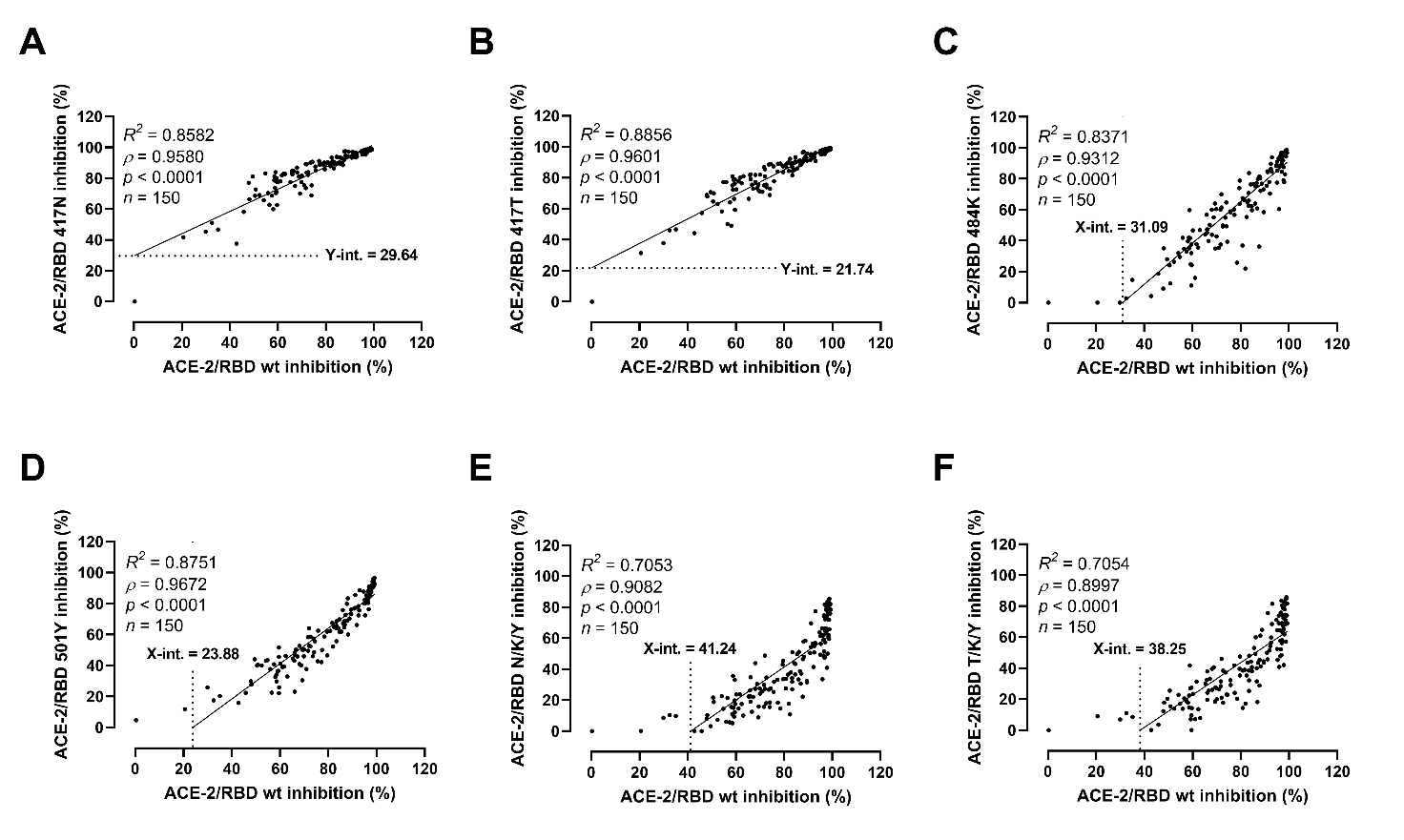
**

**Supplementary Figure 1.** Linear regression and Spearman correlation analyses of the inhibitory potency of COVID-19 convalescent patient sera for wt vs K417N **(A)**, K417T **(B)**, E484K **(C)**, N501Y **(D)**, N_K_Y (B.1.351) **(E)**, and T_K_Y (P.1) **(F)**. Dashed lines represent X and Y intersects, solid lines represent linear regression. Negative inhibition values were normalized to 0. ****, *p* < 0.0001.
